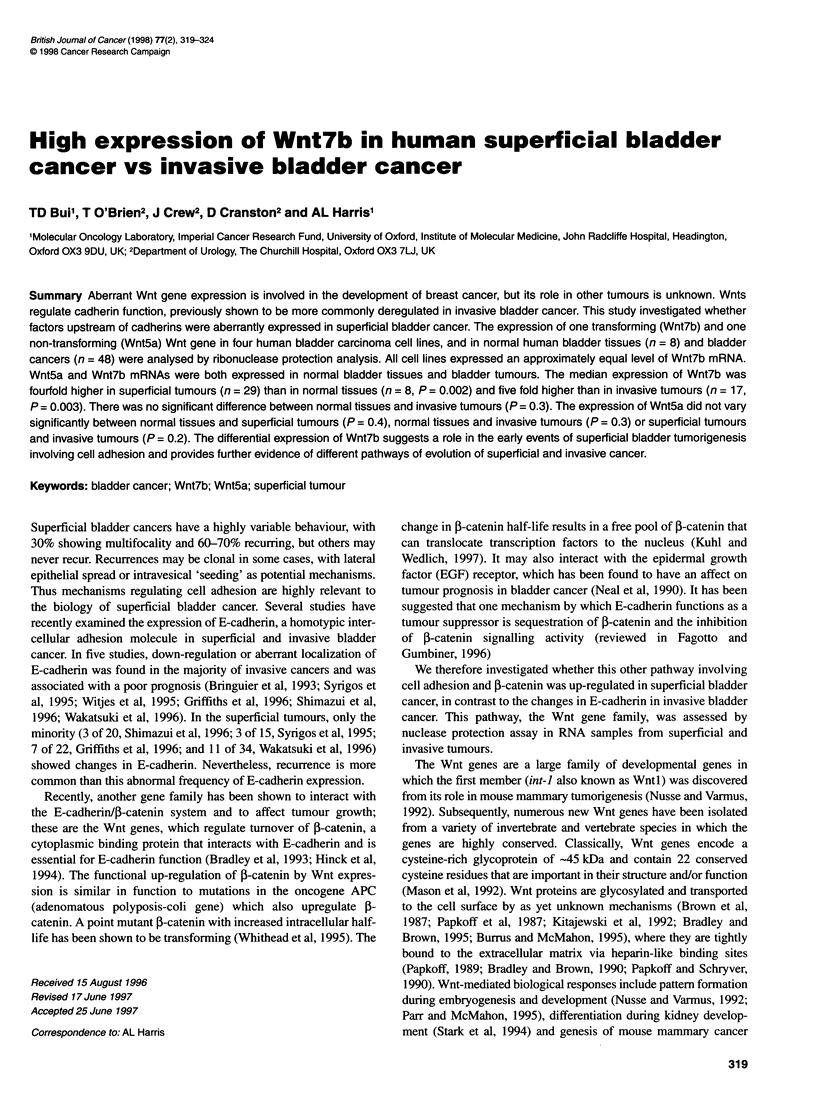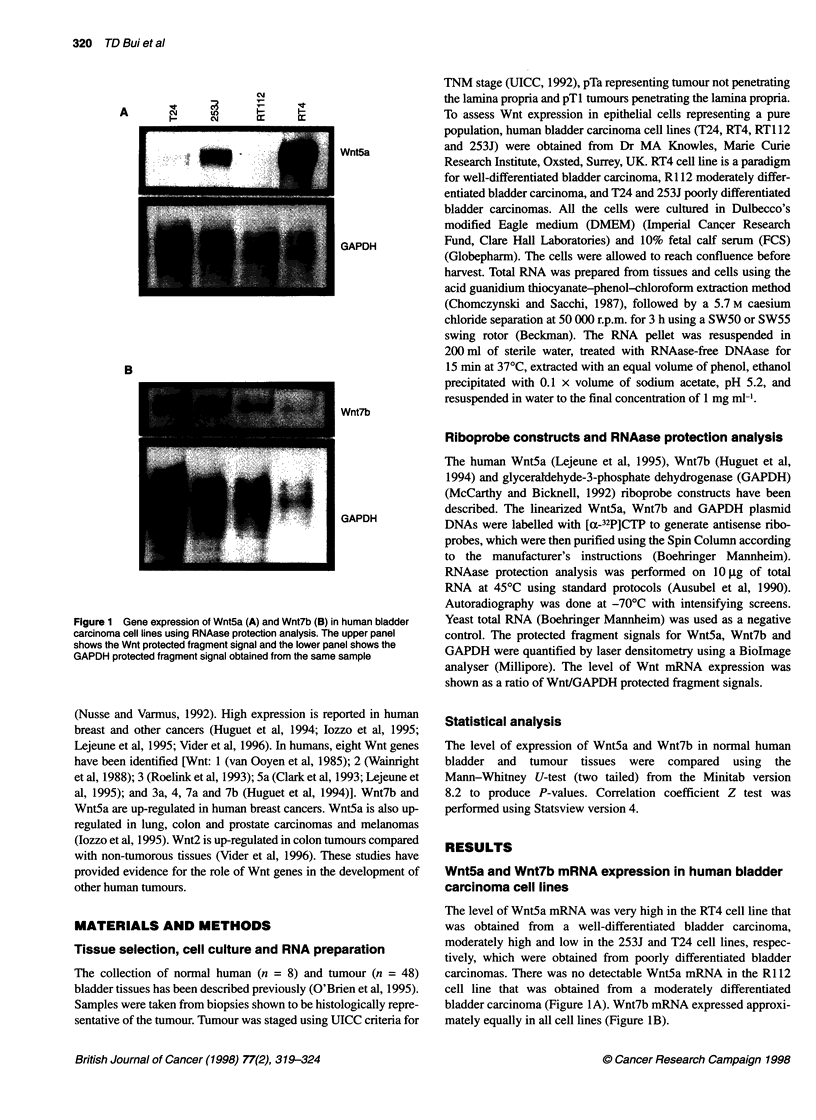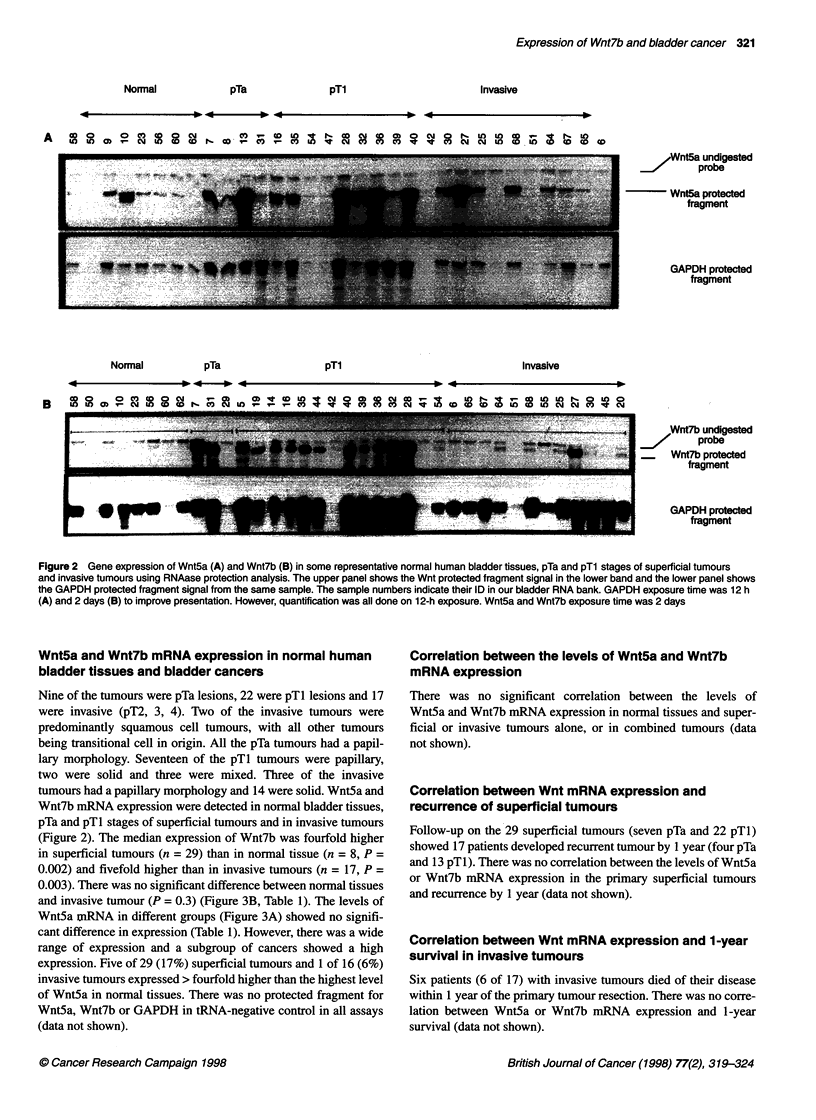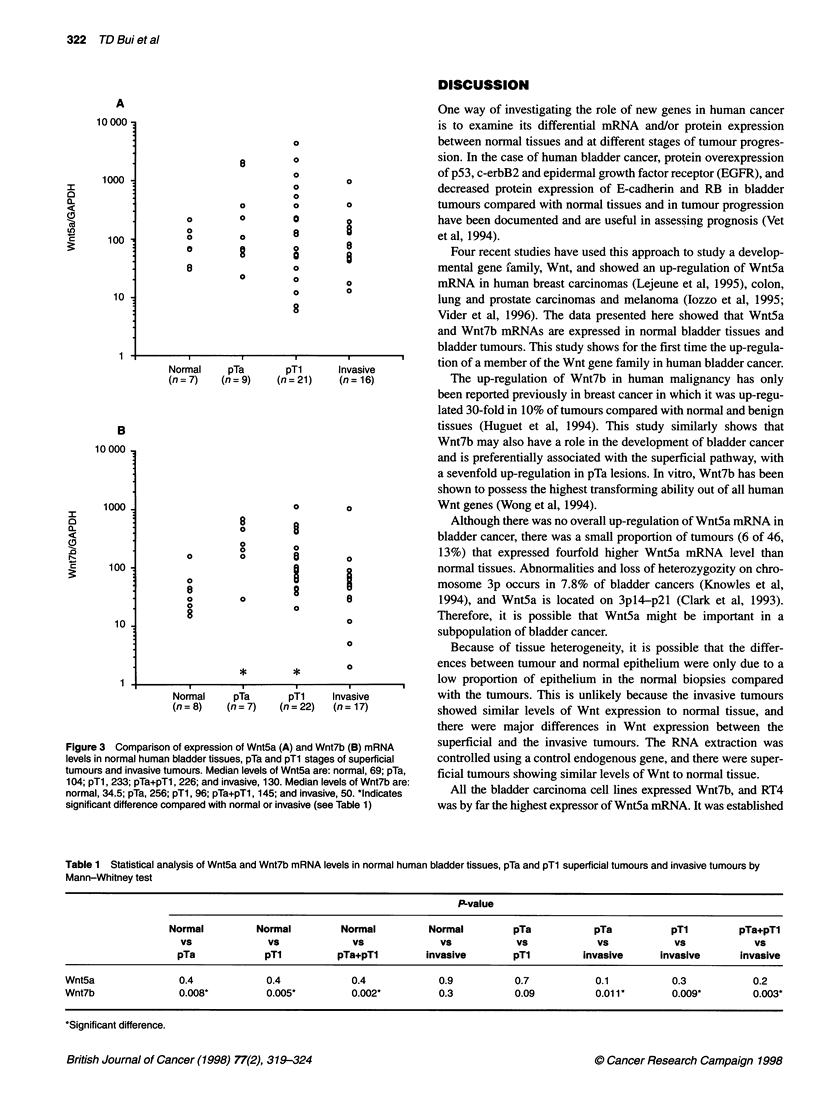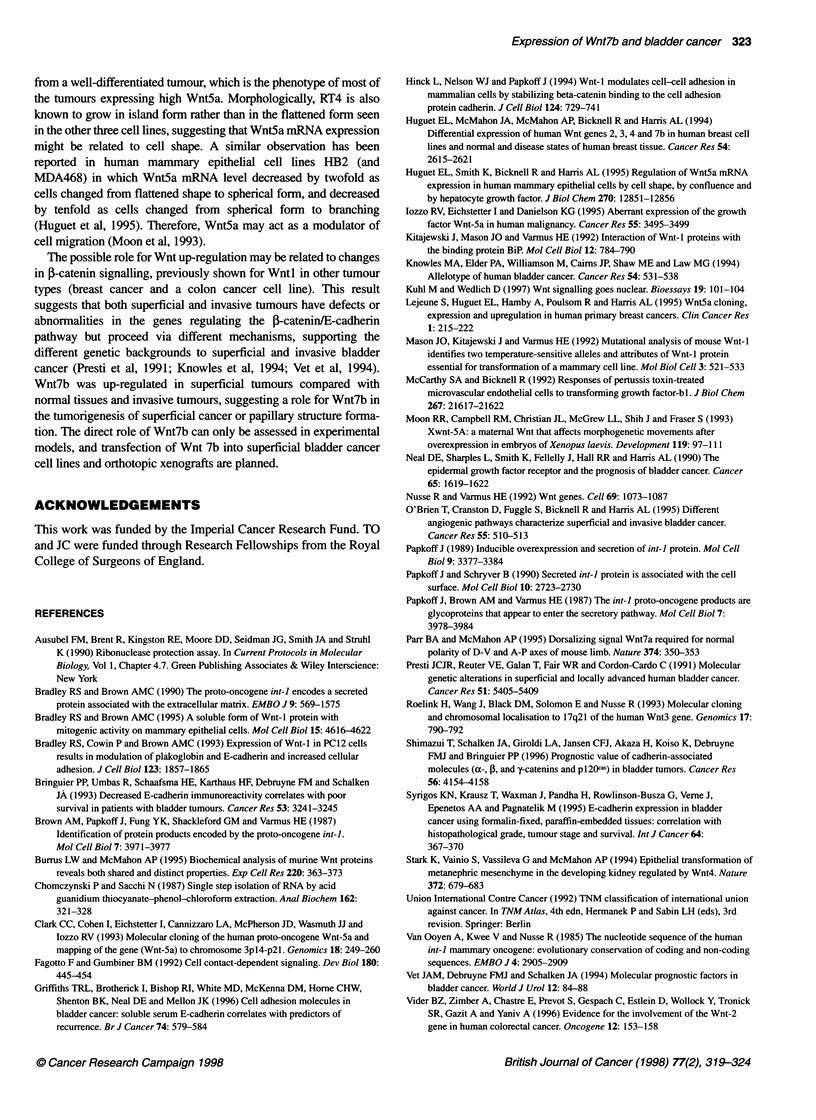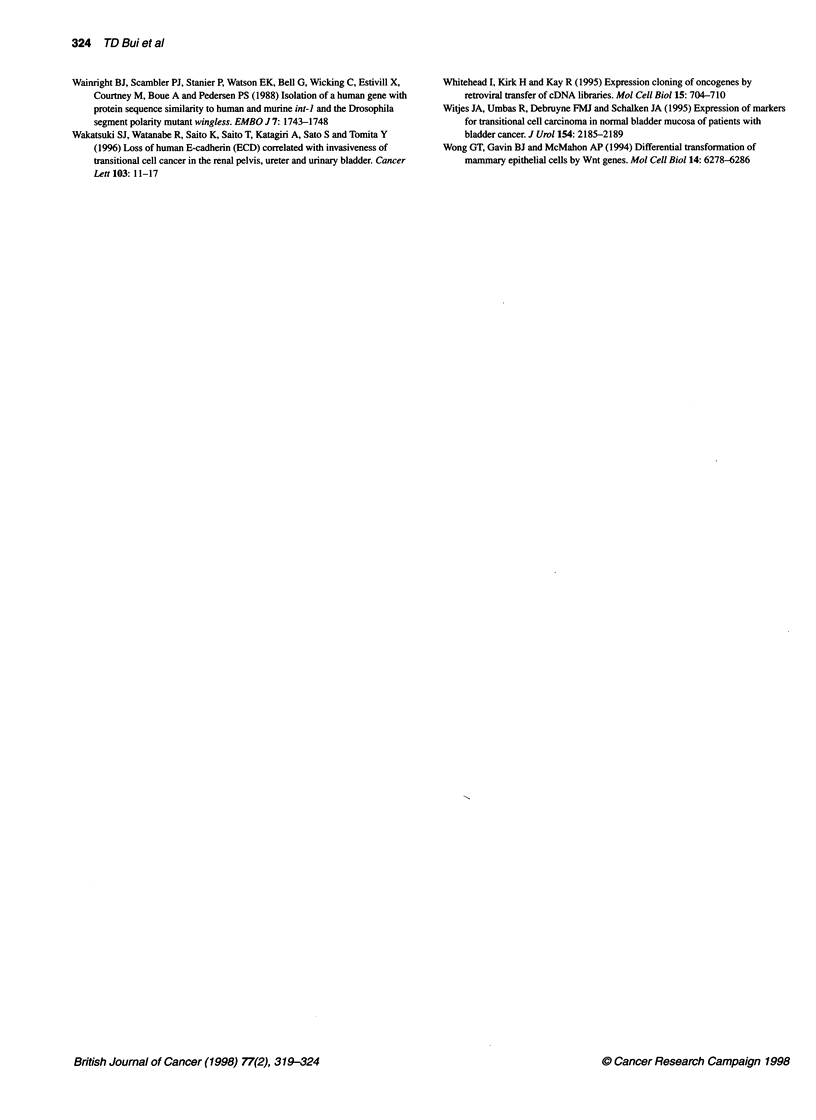# High expression of Wnt7b in human superficial bladder cancer vs invasive bladder cancer.

**DOI:** 10.1038/bjc.1998.49

**Published:** 1998

**Authors:** T. D. Bui, T. O'Brien, J. Crew, D. Cranston, A. L. Harris

**Affiliations:** Imperial Cancer Research Fund, University of Oxford, Institute of Molecular Medicine, John Radcliffe Hospital, UK.

## Abstract

**Images:**


*British Journal of Cancer* (1998) **77**(2), 319–324
© 1998 Cancer Research Campaign

### High expression of Wnt7b in human superficial bladder cancer vs invasive bladder cancer

**TD Bui[1], T O'Brien[2], J Crew[2], D Cranston[2] and AL Harris[1]**

[1]Molecular Oncology Laboratory, Imperial Cancer Research Fund, University of Oxford, Institute of Molecular Medicine, John Radcliffe Hospital, Headington, Oxford OX3 9DU, UK; [2]Department of Urology, The Churchill Hospital, Oxford OX3 7LJ, UK

**Summary** Aberrant Wnt gene expression is involved in the development of breast cancer, but its role in other tumours is unknown. Wnts regulate cadherin function, previously shown to be more commonly deregulated in invasive bladder cancer. This study investigated whether factors upstream of cadherins were aberrantly expressed in superficial bladder cancer. The expression of one transforming (Wnt7b) and one non-transforming (Wnt5a) Wnt gene in four human bladder carcinoma cell lines, and in normal human bladder tissues ($n$ = 8) and bladder cancers ($n$ = 48) were analysed by ribonuclease protection analysis. All cell lines expressed an approximately equal level of Wnt7b mRNA. Wnt5a and Wnt7b mRNAs were both expressed in normal bladder tissues and bladder tumours. The median expression of Wnt7b was fourfold higher in superficial tumours ($n$ = 29) than in normal tissues ($n$ = 8, $P$ = 0.002) and five fold higher than in invasive tumours ($n$ = 17, $P$ = 0.003). There was no significant difference between normal tissues and invasive tumours ($P$ = 0.3). The expression of Wnt5a did not vary significantly between normal tissues and superficial tumours ($P$ = 0.4), normal tissues and invasive tumours ($P$ = 0.3) or superficial tumours and invasive tumours ($P$ = 0.2). The differential expression of Wnt7b suggests a role in the early events of superficial bladder tumorigenesis involving cell adhesion and provides further evidence of different pathways of evolution of superficial and invasive cancer.

**Keywords:** bladder cancer; Wnt7b; Wnt5a; superficial tumour

Superficial bladder cancers have a highly variable behaviour, with 30% showing multifocality and 60–70% recurring, but others may never recur. Recurrences may be clonal in some cases, with lateral epithelial spread or intravesical 'seeding' as potential mechanisms. Thus mechanisms regulating cell adhesion are highly relevant to the biology of superficial bladder cancer. Several studies have recently examined the expression of E-cadherin, a homotypic intercellular adhesion molecule in superficial and invasive bladder cancer. In five studies, down-regulation or aberrant localization of E-cadherin was found in the majority of invasive cancers and was associated with a poor prognosis (Bringuier et al, 1993; Syrigos et al, 1995; Witjes et al, 1995; Griffiths et al, 1996; Shimazui et al, 1996; Wakatsuki et al, 1996). In the superficial tumours, only the minority (3 of 20, Shimazui et al, 1996; 3 of 15, Syrigos et al, 1995; 7 of 22, Griffiths et al, 1996; and 11 of 34, Wakatsuki et al, 1996) showed changes in E-cadherin. Nevertheless, recurrence is more common than this abnormal frequency of E-cadherin expression.

Recently, another gene family has been shown to interact with the E-cadherin/β-catenin system and to affect tumour growth; these are the Wnt genes, which regulate turnover of β-catenin, a cytoplasmic binding protein that interacts with E-cadherin and is essential for E-cadherin function (Bradley et al, 1993; Hinck et al, 1994). The functional up-regulation of β-catenin by Wnt expression is similar in function to mutations in the oncogene APC (adenomatous polyposis-coli gene) which also upregulate β-catenin. A point mutant β-catenin with increased intracellular half-life has been shown to be transforming (Whithead et al, 1995). The change in β-catenin half-life results in a free pool of β-catenin that can translocate transcription factors to the nucleus (Kuhl and Wedlich, 1997). It may also interact with the epidermal growth factor (EGF) receptor, which has been found to have an affect on tumour prognosis in bladder cancer (Neal et al, 1990). It has been suggested that one mechanism by which E-cadherin functions as a tumour suppressor is sequestration of β-catenin and the inhibition of β-catenin signalling activity (reviewed in Fagotto and Gumbiner, 1996)

We therefore investigated whether this other pathway involving cell adhesion and β-catenin was up-regulated in superficial bladder cancer, in contrast to the changes in E-cadherin in invasive bladder cancer. This pathway, the Wnt gene family, was assessed by nuclease protection assay in RNA samples from superficial and invasive tumours.

The Wnt genes are a large family of developmental genes in which the first member (*int-1* also known as Wnt1) was discovered from its role in mouse mammary tumorigenesis (Nusse and Varmus, 1992). Subsequently, numerous new Wnt genes have been isolated from a variety of invertebrate and vertebrate species in which the genes are highly conserved. Classically, Wnt genes encode a cysteine-rich glycoprotein of ~45 kDa and contain 22 conserved cysteine residues that are important in their structure and/or function (Mason et al, 1992). Wnt proteins are glycosylated and transported to the cell surface by as yet unknown mechanisms (Brown et al, 1987; Papkoff et al, 1987; Kitajewski et al, 1992; Bradley and Brown, 1995; Burrus and McMahon, 1995), where they are tightly bound to the extracellular matrix via heparin-like binding sites (Papkoff, 1989; Bradley and Brown, 1990; Papkoff and Schryver, 1990). Wnt-mediated biological responses include pattern formation during embryogenesis and development (Nusse and Varmus, 1992; Parr and McMahon, 1995), differentiation during kidney development (Stark et al, 1994) and genesis of mouse mammary cancer

*Received 15 August 1996*
*Revised 17 June 1997*
*Accepted 25 June 1997*

*Correspondence to:* AL Harris

**Figure 1** Gene expression of Wnt5a (**A**) and Wnt7b (**B**) in human bladder carcinoma cell lines using RNAase protection analysis. The upper panel shows the Wnt protected fragment signal and the lower panel shows the GAPDH protected fragment signal obtained from the same sample

(Nusse and Varmus, 1992). High expression is reported in human breast and other cancers (Huguet et al, 1994; Iozzo et al, 1995; Lejeune et al, 1995; Vider et al, 1996). In humans, eight Wnt genes have been identified [Wnt: 1 (van Ooyen et al, 1985); 2 (Wainright et al, 1988); 3 (Roelink et al, 1993); 5a (Clark et al, 1993; Lejeune et al, 1995); and 3a, 4, 7a and 7b (Huguet et al, 1994)]. Wnt7b and Wnt5a are up-regulated in human breast cancers. Wnt5a is also up-regulated in lung, colon and prostate carcinomas and melanomas (Iozzo et al, 1995). Wnt2 is up-regulated in colon tumours compared with non-tumorous tissues (Vider et al, 1996). These studies have provided evidence for the role of Wnt genes in the development of other human tumours.

### MATERIALS AND METHODS

### Tissue selection, cell culture and RNA preparation

The collection of normal human ($n$ = 8) and tumour ($n$ = 48) bladder tissues has been described previously (O'Brien et al, 1995). Samples were taken from biopsies shown to be histologically representative of the tumour. Tumour was staged using UICC criteria for TNM stage (UICC, 1992), pTa representing tumour not penetrating the lamina propria and pT1 tumours penetrating the lamina propria. To assess Wnt expression in epithelial cells representing a pure population, human bladder carcinoma cell lines (T24, RT4, RT112 and 253J) were obtained from Dr MA Knowles, Marie Curie Research Institute, Oxsted, Surrey, UK. RT4 cell line is a paradigm for well-differentiated bladder carcinoma, R112 moderately differentiated bladder carcinoma, and T24 and 253J poorly differentiated bladder carcinomas. All the cells were cultured in Dulbecco's modified Eagle medium (DMEM) (Imperial Cançer Research Fund, Clare Hall Laboratories) and 10% fetal calf serum (FCS) (Globepharm). The cells were allowed to reach confluence before harvest. Total RNA was prepared from tissues and cells using the acid guanidium thiocyanate–phenol–chloroform extraction method (Chomczynski and Sacchi, 1987), followed by a 5.7 M caesium chloride separation at 50 000 r.p.m. for 3 h using a SW50 or SW55 swing rotor (Beckman). The RNA pellet was resuspended in 200 ml of sterile water, treated with RNAase-free DNAase for 15 min at 37°C, extracted with an equal volume of phenol, ethanol precipitated with 0.1 × volume of sodium acetate, pH 5.2, and resuspended in water to the final concentration of 1 mg $ml^{-1}$.

### Riboprobe constructs and RNAase protection analysis

The human Wnt5a (Lejeune et al, 1995), Wnt7b (Huguet et al, 1994) and glyceraldehyde-3-phosphate dehydrogenase (GAPDH) (McCarthy and Bicknell, 1992) riboprobe constructs have been described. The linearized Wnt5a, Wnt7b and GAPDH plasmid DNAs were labelled with [$\alpha$-$^{32}$P]CTP to generate antisense riboprobes, which were then purified using the Spin Column according to the manufacturer's instructions (Boehringer Mannheim). RNAase protection analysis was performed on 10 μg of total RNA at 45°C using standard protocols (Ausubel et al, 1990). Autoradiography was done at –70°C with intensifying screens. Yeast total RNA (Boehringer Mannheim) was used as a negative control. The protected fragment signals for Wnt5a, Wnt7b and GAPDH were quantified by laser densitometry using a BioImage analyser (Millipore). The level of Wnt mRNA expression was shown as a ratio of Wnt/GAPDH protected fragment signals.

### Statistical analysis

The level of expression of Wnt5a and Wnt7b in normal human bladder and tumour tissues were compared using the Mann–Whitney $U$-test (two tailed) from the Minitab version 8.2 to produce $P$-values. Correlation coefficient $Z$ test was performed using Statsview version 4.

### RESULTS

### Wnt5a and Wnt7b mRNA expression in human bladder carcinoma cell lines

The level of Wnt5a mRNA was very high in the RT4 cell line that was obtained from a well-differentiated bladder carcinoma, moderately high and low in the 253J and T24 cell lines, respectively, which were obtained from poorly differentiated bladder carcinomas. There was no detectable Wnt5a mRNA in the R112 cell line that was obtained from a moderately differentiated bladder carcinoma (Figure 1A). Wnt7b mRNA expressed approximately equally in all cell lines (Figure 1B).

© Cancer Research Campaign 1998

**Figure 2** Gene expression of Wnt5a (**A**) and Wnt7b (**B**) in some representative normal human bladder tissues, pTa and pT1 stages of superficial tumours and invasive tumours using RNAase protection analysis. The upper panel shows the Wnt protected fragment signal in the lower band and the lower panel shows the GAPDH protected fragment signal from the same sample. The sample numbers indicate their ID in our bladder RNA bank. GAPDH exposure time was 12 h (**A**) and 2 days (**B**) to improve presentation. However, quantification was all done on 12-h exposure. Wnt5a and Wnt7b exposure time was 2 days

### Wnt5a and Wnt7b mRNA expression in normal human bladder tissues and bladder cancers

Nine of the tumours were pTa lesions, 22 were pT1 lesions and 17 were invasive (pT2, 3, 4). Two of the invasive tumours were predominantly squamous cell tumours, with all other tumours being transitional cell in origin. All the pTa tumours had a papillary morphology. Seventeen of the pT1 tumours were papillary, two were solid and three were mixed. Three of the invasive tumours had a papillary morphology and 14 were solid. Wnt5a and Wnt7b mRNA expression were detected in normal bladder tissues, pTa and pT1 stages of superficial tumours and in invasive tumours (Figure 2). The median expression of Wnt7b was fourfold higher in superficial tumours ($n$ = 29) than in normal tissue ($n$ = 8, $P$ = 0.002) and fivefold higher than in invasive tumours ($n$ = 17, $P$ = 0.003). There was no significant difference between normal tissues and invasive tumour ($P$ = 0.3) (Figure 3B, Table 1). The levels of Wnt5a mRNA in different groups (Figure 3A) showed no significant difference in expression (Table 1). However, there was a wide range of expression and a subgroup of cancers showed a high expression. Five of 29 (17%) superficial tumours and 1 of 16 (6%) invasive tumours expressed > fourfold higher than the highest level of Wnt5a in normal tissues. There was no protected fragment for Wnt5a, Wnt7b or GAPDH in tRNA-negative control in all assays (data not shown).

### Correlation between the levels of Wnt5a and Wnt7b mRNA expression

There was no significant correlation between the levels of Wnt5a and Wnt7b mRNA expression in normal tissues and superficial or invasive tumours alone, or in combined tumours (data not shown).

### Correlation between Wnt mRNA expression and recurrence of superficial tumours

Follow-up on the 29 superficial tumours (seven pTa and 22 pT1) showed 17 patients developed recurrent tumour by 1 year (four pTa and 13 pT1). There was no correlation between the levels of Wnt5a or Wnt7b mRNA expression in the primary superficial tumours and recurrence by 1 year (data not shown).

### Correlation between Wnt mRNA expression and 1-year survival in invasive tumours

Six patients (6 of 17) with invasive tumours died of their disease within 1 year of the primary tumour resection. There was no correlation between Wnt5a or Wnt7b mRNA expression and 1-year survival (data not shown).

© Cancer Research Campaign 1998

**Figure 3** Comparison of expression of Wnt5a (**A**) and Wnt7b (**B**) mRNA levels in normal human bladder tissues, pTa and pT1 stages of superficial tumours and invasive tumours. Median levels of Wnt5a are: normal, 69; pTa, 104; pT1, 233; pTa+pT1, 226; and invasive, 130. Median levels of Wnt7b are: normal, 34.5; pTa, 256; pT1, 96; pTa+pT1, 145; and invasive, 50. *Indicates significant difference compared with normal or invasive (see Table 1)

### DISCUSSION

One way of investigating the role of new genes in human cancer is to examine its differential mRNA and/or protein expression between normal tissues and at different stages of tumour progression. In the case of human bladder cancer, protein overexpression of p53, c-erbB2 and epidermal growth factor receptor (EGFR), and decreased protein expression of E-cadherin and RB in bladder tumours compared with normal tissues and in tumour progression have been documented and are useful in assessing prognosis (Vet et al, 1994).

Four recent studies have used this approach to study a developmental gene family, Wnt, and showed an up-regulation of Wnt5a mRNA in human breast carcinomas (Lejeune et al, 1995), colon, lung and prostate carcinomas and melanoma (Iozzo et al, 1995; Vider et al, 1996). The data presented here showed that Wnt5a and Wnt7b mRNAs are expressed in normal bladder tissues and bladder tumours. This study shows for the first time the up-regulation of a member of the Wnt gene family in human bladder cancer.

The up-regulation of Wnt7b in human malignancy has only been reported previously in breast cancer in which it was up-regulated 30-fold in 10% of tumours compared with normal and benign tissues (Huguet et al, 1994). This study similarly shows that Wnt7b may also have a role in the development of bladder cancer and is preferentially associated with the superficial pathway, with a sevenfold up-regulation in pTa lesions. In vitro, Wnt7b has been shown to possess the highest transforming ability out of all human Wnt genes (Wong et al, 1994).

Although there was no overall up-regulation of Wnt5a mRNA in bladder cancer, there was a small proportion of tumours (6 of 46, 13%) that expressed fourfold higher Wnt5a mRNA level than normal tissues. Abnormalities and loss of heterozygozity on chromosome 3p occurs in 7.8% of bladder cancers (Knowles et al, 1994), and Wnt5a is located on 3p14–p21 (Clark et al, 1993). Therefore, it is possible that Wnt5a might be important in a subpopulation of bladder cancer.

Because of tissue heterogeneity, it is possible that the differences between tumour and normal epithelium were only due to a low proportion of epithelium in the normal biopsies compared with the tumours. This is unlikely because the invasive tumours showed similar levels of Wnt expression to normal tissue, and there were major differences in Wnt expression between the superficial and the invasive tumours. The RNA extraction was controlled using a control endogenous gene, and there were superficial tumours showing similar levels of Wnt to normal tissue.

All the bladder carcinoma cell lines expressed Wnt7b, and RT4 was by far the highest expressor of Wnt5a mRNA. It was established

**Table 1** Statistical analysis of Wnt5a and Wnt7b mRNA levels in normal human bladder tissues, pTa and pT1 superficial tumours and invasive tumours by Mann–Whitney test

| | *P*-value | | | | | | | |
|---|---|---|---|---|---|---|---|---|
| | Normal vs pTa | Normal vs pT1 | Normal vs pTa+pT1 | Normal vs invasive | pTa vs pT1 | pTa vs invasive | pT1 vs invasive | pTa+pT1 vs invasive |
| Wnt5a | 0.4 | 0.4 | 0.4 | 0.9 | 0.7 | 0.1 | 0.3 | 0.2 |
| Wnt7b | 0.008* | 0.005* | 0.002* | 0.3 | 0.09 | 0.011* | 0.009* | 0.003* |

*Significant difference.

 © Cancer Research Campaign 1998

from a well-differentiated tumour, which is the phenotype of most of the tumours expressing high Wnt5a. Morphologically, RT4 is also known to grow in island form rather than in the flattened form seen in the other three cell lines, suggesting that Wnt5a mRNA expression might be related to cell shape. A similar observation has been reported in human mammary epithelial cell lines HB2 (and MDA468) in which Wnt5a mRNA level decreased by twofold as cells changed from flattened shape to spherical form, and decreased by tenfold as cells changed from spherical form to branching (Huguet et al, 1995). Therefore, Wnt5a may act as a modulator of cell migration (Moon et al, 1993).

The possible role for Wnt up-regulation may be related to changes in β-catenin signalling, previously shown for Wnt1 in other tumour types (breast cancer and a colon cancer cell line). This result suggests that both superficial and invasive tumours have defects or abnormalities in the genes regulating the β-catenin/E-cadherin pathway but proceed via different mechanisms, supporting the different genetic backgrounds to superficial and invasive bladder cancer (Presti et al, 1991; Knowles et al, 1994; Vet et al, 1994). Wnt7b was up-regulated in superficial tumours compared with normal tissues and invasive tumours, suggesting a role for Wnt7b in the tumorigenesis of superficial cancer or papillary structure formation. The direct role of Wnt7b can only be assessed in experimental models, and transfection of Wnt 7b into superficial bladder cancer cell lines and orthotopic xenografts are planned.

### ACKNOWLEDGEMENTS

This work was funded by the Imperial Cancer Research Fund. TO and JC were funded through Research Fellowships from the Royal College of Surgeons of England.

### REFERENCES

Ausubel FM, Brent R, Kingston RE, Moore DD, Seidman JG, Smith JA and Struhl K (1990) Ribonuclease protection assay. In *Current Protocols in Molecular Biology*, Vol 1, Chapter 4.7. Green Publishing Associates & Wiley Interscience: New York

Bradley RS and Brown AMC (1990) The proto-oncogene *int-1* encodes a secreted protein associated with the extracellular matrix. *EMBO J* **9**: 569–1575

Bradley RS and Brown AMC (1995) A soluble form of Wnt-1 protein with mitogenic activity on mammary epithelial cells. *Mol Cell Biol* **15**: 4616–4622

Bradley RS, Cowin P and Brown AMC (1993) Expression of Wnt-1 in PC12 cells results in modulation of plakoglobin and E-cadherin and increased cellular adhesion. *J Cell Biol* **123**: 1857–1865

Bringuier PP, Umbas R, Schaafsma HE, Karthaus HF, Debruyne FM and Schalken JÀ (1993) Decreased E-cadherin immunoreactivity correlates with poor survival in patients with bladder tumours. *Cancer Res* **53**: 3241–3245

Brown AM, Papkoff J, Fung YK, Shackleford GM and Varmus HE (1987) Identification of protein products encoded by the proto-oncogene *int-1*. *Mol Cell Biol* **7**: 3971–3977

Burrus LW and McMahon AP (1995) Biochemical analysis of murine Wnt proteins reveals both shared and distinct properties. *Exp Cell Res* **220**: 363–373

Chomczynski P and Sacchi N (1987) Single step isolation of RNA by acid guanidium thiocyanate–phenol–chloroform extraction. *Anal Biochem* **162**: 321–328

Clark CC, Cohen I, Eichstetter I, Cannizzaro LA, McPherson JD, Wasmuth JJ and Iozzo RV (1993) Molecular cloning of the human proto-oncogene Wnt-5a and mapping of the gene (Wnt-5a) to chromosome 3p14-p21. *Genomics* **18**: 249–260

Fagotto F and Gumbiner BM (1992) Cell contact-dependent signaling. *Dev Biol* **180**: 445–454

Griffiths TRL, Brotherick I, Bishop RI, White MD, McKenna DM, Horne CHW, Shenton BK, Neal DE and Mellon JK (1996) Cell adhesion molecules in bladder cancer: soluble serum E-cadherin correlates with predictors of recurrence. *Br J Cancer* **74**: 579–584

Hinck L, Nelson WJ and Papkoff J (1994) Wnt-1 modulates cell–cell adhesion in mammalian cells by stabilizing beta-catenin binding to the cell adhesion protein cadherin. *J Cell Biol* **124**: 729–741

Huguet EL, McMahon JA, McMahon AP, Bicknell R and Harris AL (1994) Differential expression of human Wnt genes 2, 3, 4 and 7b in human breast cell lines and normal and disease states of human breast tissue. *Cancer Res* **54**: 2615–2621

Huguet EL, Smith K, Bicknell R and Harris AL (1995) Regulation of Wnt5a mRNA expression in human mammary epithelial cells by cell shape, by confluence and by hepatocyte growth factor. *J Biol Chem* **270**: 12851–12856

Iozzo RV, Eichstetter I and Danielson KG (1995) Aberrant expression of the growth factor Wnt-5a in human malignancy. *Cancer Res* **55**: 3495–3499

Kitajewski J, Mason JO and Varmus HE (1992) Interaction of Wnt-1 proteins with the binding protein BiP. *Mol Cell Biol* **12**: 784–790

Knowles MA, Elder PA, Williamson M, Cairns JP, Shaw ME and Law MG (1994) Allelotype of human bladder cancer. *Cancer Res* **54**: 531–538

Kuhl M and Wedlich D (1997) Wnt signalling goes nuclear. *Bioessays* **19**: 101–104

Lejeune S, Huguet EL, Hamby A, Poulsom R and Harris AL (1995) Wnt5a cloning, expression and upregulation in human primary breast cancers. *Clin Cancer Res* **1**: 215–222

Mason JO, Kitajewski J and Varmus HE (1992) Mutational analysis of mouse Wnt-1 identifies two temperature-sensitive alleles and attributes of Wnt-1 protein essential for transformation of a mammary cell line. *Mol Biol Cell* **3**: 521–533

McCarthy SA and Bicknell R (1992) Responses of pertussis toxin-treated microvascular endothelial cells to transforming growth factor-b1. *J Biol Chem* **267**: 21617–21622

Moon RR, Campbell RM, Christian JL, McGrew LL, Shih J and Fraser S (1993) Xwnt-5A: a maternal Wnt that affects morphogenetic movements after overexpression in embryos of *Xenopus laevis*. *Development* **119**: 97–111

Neal DE, Sharples L, Smith K, Fellelly J, Hall RR and Harris AL (1990) The epidermal growth factor receptor and the prognosis of bladder cancer. *Cancer* **65**: 1619–1622

Nusse R and Varmus HE (1992) Wnt genes. *Cell* **69**: 1073–1087

O'Brien T, Cranston D, Fuggle S, Bicknell R and Harris AL (1995) Different angiogenic pathways characterize superficial and invasive bladder cancer. *Cancer Res* **55**: 510–513

Papkoff J (1989) Inducible overexpression and secretion of *int-1* protein. *Mol Cell Biol* **9**: 3377–3384

Papkoff J and Schryver B (1990) Secreted *int-1* protein is associated with the cell surface. *Mol Cell Biol* **10**: 2723–2730

Papkoff J, Brown AM and Varmus HE (1987) The *int-1* proto-oncogene products are glycoproteins that appear to enter the secretory pathway. *Mol Cell Biol* **7**: 3978–3984

Parr BA and McMahon AP (1995) Dorsalizing signal Wnt7a required for normal polarity of D-V and A-P axes of mouse limb. *Nature* **374**: 350–353

Presti JCJR, Reuter VE, Galan T, Fair WR and Cordon-Cardo C (1991) Molecular genetic alterations in superficial and locally advanced human bladder cancer. *Cancer Res* **51**: 5405–5409

Roelink H, Wang J, Black DM, Solomon E and Nusse R (1993) Molecular cloning and chromosomal localisation to 17q21 of the human Wnt3 gene. *Genomics* **17**: 790–792

Shimazui T, Schalken JA, Giroldi LA, Jansen CFJ, Akaza H, Koiso K, Debruyne FMJ and Bringuier PP (1996) Prognostic value of cadherin-associated molecules (α-, β, and γ-catenins and p120$^{cas}$) in bladder tumors. *Cancer Res* **56**: 4154–4158

Syrigos KN, Krausz T, Waxman J, Pandha H, Rowlinson-Busza G, Verne J, Epenetos AA and Pagnatelik M (1995) E-cadherin expression in bladder cancer using formalin-fixed, paraffin-embedded tissues: correlation with histopathological grade, tumour stage and survival. *Int J Cancer* **64**: 367–370

Stark K, Vainio S, Vassileva G and McMahon AP (1994) Epithelial transformation of metanephric mesenchyme in the developing kidney regulated by Wnt4. *Nature* **372**: 679–683

Union International Contre Cancer (1992) TNM classification of international union against cancer. In *TNM Atlas*, 4th edn, Hermanek P and Sabin LH (eds), 3rd revision. Springer: Berlin

Van Ooyen A, Kwee V and Nusse R (1985) The nucleotide sequence of the human *int-1* mammary oncogene: evolutionary conservation of coding and non-coding sequences. *EMBO J* **4**: 2905–2909

Vet JAM, Debruyne FMJ and Schalken JA (1994) Molecular prognostic factors in bladder cancer. *World J Urol* **12**: 84–88

Vider BZ, Zimber A, Chastre E, Prevot S, Gespach C, Estlein D, Wollock Y, Tronick SR, Gazit A and Yaniv A (1996) Evidence for the involvement of the Wnt-2 gene in human colorectal cancer. *Oncogene* **12**: 153–158

© Cancer Research Campaign 1998 

Wainright BJ, Scambler PJ, Stanier P, Watson EK, Bell G, Wicking C, Estivill X, Courtney M, Boue A and Pedersen PS (1988) Isolation of a human gene with protein sequence similarity to human and murine *int-1* and the Drosophila segment polarity mutant *wingless*. *EMBO J* **7**: 1743–1748

Wakatsuki SJ, Watanabe R, Saito K, Saito T, Katagiri A, Sato S and Tomita Y (1996) Loss of human E-cadherin (ECD) correlated with invasiveness of transitional cell cancer in the renal pelvis, ureter and urinary bladder. *Cancer Lett* **103**: 11–17

Whitehead I, Kirk H and Kay R (1995) Expression cloning of oncogenes by retroviral transfer of cDNA libraries. *Mol Cell Biol* **15**: 704–710

Witjes JA, Umbas R, Debruyne FMJ and Schalken JA (1995) Expression of markers for transitional cell carcinoma in normal bladder mucosa of patients with bladder cancer. *J Urol* **154**: 2185–2189

Wong GT, Gavin BJ and McMahon AP (1994) Differential transformation of mammary epithelial cells by Wnt genes. *Mol Cell Biol* **14**: 6278–6286

*© Cancer Research Campaign 1998*